# An Innovative Nanobody-Based High-Biocompatibility Gold Interdigitated Microelectrode Electrochemical Bioimpedance Sensor for the Ultrasensitive Detection of Difenacoum in Human Serum

**DOI:** 10.3390/ma14143930

**Published:** 2021-07-14

**Authors:** Liuchuan Guo, Sihan Wang, Zhiwei He, Jing Zhang, Xiaoli Zhu, Yuebin Ke, Haiyang Jiang, Zhanhui Wang

**Affiliations:** 1 College of Veterinary Medicine, China Agricultural University, Beijing Key Laboratory of Detection Technology for Animal-Derived Food, Beijing Laboratory for Food Quality and Safety, Beijing 100193, China; bs20183050440@cau.edu.cn (L.G.); wangshjaz@cau.edu.cn (S.W.); 2 Department of Applied Physics, China Agricultural University, Beijing 100083, China; hezw@cau.edu.cn; 3 College of Electronic and Information Engineering, North China University of Technology, Beijing 100144, China; zhangjing@ncut.edu.cn; 4 Key Laboratory of Microelectronics Devices & Integrated Technology, Institute of Microelectronics, Chinese Academy of Sciences, Beijing 100029, China; zhuxiaoli@ime.ac.cn; 5 Key Laboratory of Molecular Epidemiology of Shenzhen, Shenzhen Center for Disease Control and Prevention, Shenzhen 518020, China; keyke@szu.edu.cn

**Keywords:** difenacoum, nanobody, polycrystalline electrode, bioimpedance sensor

## Abstract

Difenacoum (DIF) is one of the most widely used anticoagulant rodenticides. However, accidental or intentional ingestion of DIF seriously threatens humans and other non-target species. Therefore, a rapid and sensitive detection method to quantify DIF is urgently needed. In this study, one anti-DIF nanobody (Nb) was assembled on the surface of a gold interdigitated microelectrode (IDME) using an Au–S bond to fabricate a bioimpedance sensor. To improve the immobilization amount of Nbs on the electrode, a polycrystalline gold IDME was prepared to provide a larger surface and better biocompatibility. Thus, a novel and ultrasensitive bioimpedance sensor based on electrochemical impedance spectroscopy (EIS) was designed for the determination of DIF, and it displayed good reproducibility and stability in human serum. The proposed bioimpedance sensor displayed a wide working range, between 0.1–1000 pg/mL, with a limit of detection (LOD) of 0.1 pg/mL of DIF. This method exhibited excellent performance, good sensitivity, and reproducibility and achieved the highest sensitivity of all currently existing methods used to quantify DIF. The highly sensitive DIF detection of this proposed bioimpedance sensor indicates its potential as an efficacious approach for DIF monitoring in human serum with high accuracy and precision.

## 1. Introduction

DIF (3-(3-biphenyl-4-yl-1,2,3,4-tetrahydro-l-naphthyl)-4-hydroxycoumarin), a derivative of 4-hydroxycoumarin, is a second-generation anticoagulant rodenticide that has been used for decades worldwide as a form of rodent control [1,2]. In 1975, DIF was developed amidst increasing concern about warfarin-resistant rats [3]. The anticoagulant activity of DIF is more effective and toxic than the first-generation anticoagulant rodenticides, such as warfarin, because of the introduction of substituted tetralinyl on the side chain [4,5]. DIF can act as an anti-vitamin K anticoagulant to block the vitamin K cycle, which stops the activity of the vitamin K-dependent clotting factor and impairs the biosynthesis of a variety of coagulation functions [6]. DIF can also damage the capillary walls, leading to hemorrhages, necrosis of the parenchymal organs, and even death [7]. The LD_50_-value (lethal dose for 50% of the test animals) for DIF is 1.8 mg/kg in rats and 2.0 mg/kg in rabbits for oral ingestion [8]. The use of DIF has reached its highest proportion (28.3%) as an active substance among registered products in Germany [9] and can be found in approximately 45% of agricultural premises in Great Britain, making it the most wildly used anticoagulant rodenticide globally [10]. As DIF has been widely used for many years and is highly accessible, it acts as a poison in both target and non-target animals, including humans [11,12]. The most common forms of humans poisoned by DIF consist of accidental ingestion, human poisoning, suicide attempts and secondary exposure. Even more worrying is that DIF has a slow metabolism and an extremely long half-life in the human body [13]. The period of harmfulness for DIF in cases of human poisoning has been found to range from 10 days to a few months [4]. Hence, it is important to establish rapid and ultra-sensitive detection methods for DIF for the purpose of early diagnosis and treatment.

Currently, the primary detection methods for DIF primarily rely on exactitude apparatuses, such as liquid chromatography–tandem mass spectrometry (LC–MS/MS) and high-performance liquid chromatography (HPLC) [11,14]. However, these methods are time-consuming and require precise equipment and professional operators, which remains a significant limitation. Consequently, it is essential to develop alternative approaches for DIF detection. An immunoassay, as a rapid and sensitive method based on the specific recognition of antigens and antibodies, is widely applied in many fields, especially for diagnosis and detection. The lateral flow immunoassay (LFIA), a conventional and well-established immunoassay, as the most popular point-of-care diagnostic tool in many fields, has been applied for DIF [15]. However, LFIA generally has a low sensitivity relative to other immunological methods. Hence, a highly sensitive immunoassay is necessary, especially when the analyte is required to be detected at a low concentration. Furthermore, electrochemical immunoassay sensors offer time savings, cost savings, the visible results and sensitivity of electrochemistry, and the high specificity of immunoassays, which are increasingly used for small molecular detection [16,17]. Little attention had been focused on the development of electrochemical immunoassays for DIF. Electrochemical impedance spectroscopy (EIS) can directly relate the impedance change to the concentration of the analytes by observing the impedance signal changes of the adsorbed targets to the functionalized electrodes. The interdigitated microelectrode (IDME), as a classic impedance sensor, has been applied in several highly sensitive biosensing applications [18,19]. Currently, immunosensors developed based on IDME are affected by structure and size and often have the defects of low bio-affinity and poor antibody coupling effects. Hence, the appropriate design and optimization of IDME is crucial for the detection performance. In addition, Au has been widely applied for IDME construction due to the simple fabrication and ease in forming self-assembled monolayers [20]. To pursue a higher sensitivity of a bioimpedance sensor, it is necessary to increase the amount of antibody as much as possible on the surface of an electrode. In this work, based on our previous research [21], an IDME with the interdigitated gap of 5 μm is fabricated, and an immunosensor is prepared using self-assembled antibodies on the electrode surface. The polycrystalline gold IDME shows a larger surface roughness of the gold film on the electrode than the single-crystal gold IDME and achieve a high coupling rate of the electrode to the antibody.

Antibodies are one of the most significant receptors to target specific analytes, making use of the highly specific interaction of the antibody and antigen. In the 1990s, new antibodies, called heavy-chain-only antibodies (HcAbs), were first discovered in camelidae [22]. Unlike the conventional immunoglobulin G (IgG), HcAbs are devoid of light chains and only include two constant domains (CH_2_ and CH_3_), a hinge region and a variable heavy-chain domain (VHH) [23]. The isolated VHHs domain, also known as Nbs or single-domain antibodies (sdAbs), retains full antigen-binding capacity and comprises only a single variable domain, but is functionally equivalent to full-length traditional antibodies [24]. Nbs are the smallest antibodies that are known, and their essential characteristic is their small size (diameter and height of 2.5 nm and 4 nm, respectively, and approximately 15 kDa) [25]. Compared to other types of antibodies, Nbs can remain active under harsh conditions, like extreme temperatures, organic solvents, or ultra pH, which equip them with better stability for applications [24,26]. Compared to traditional monoclonal antibodies (about 150 kDa), Nbs can increases the density of antibody on the surface of the sensor, which display a lower limit of detection (LOD) of the analyte. Nowadays, Nbs have been useful for the detection of bacterial, viruses, toxins, and other antigens. There have been some studies of electrochemical detection assays based on Nb-modified electrodes [27,28]. In this work, an innovative approach is conceived based on a combination of Nbs and IDME to open new opportunities for the detection of DIF.

In this technique, the IDME is surface-modified through self-assembly using the anti-DIF Nb AR-H2-1 as a bio-recognition element to form a stable Au–S covalent bond on to a gold electrode. Subsequently, to increase the amount of Nbs on the electrode surface, a polycrystalline golden electrode and a single-crystal gold electrode are employed, and the degree of the Nb coupling rate with different crystal orientations on the gold electrode interface is explored. Finally, the results indicated that the bioimpedance sensor based on Nbs and polycrystalline gold IDME can be an attractive analytical tool that offers a promising approach for the determination of DIF in human serum samples. In addition, the proposed bioimpedance sensor based on the polycrystalline gold electrode is more sensitive and provides a lower LOD than that found in other published studies.

## 2. Materials and Methods

### 2.1. Reagents and Apparatuses

Potassium ferricyanide (K_3_FeC_6_N_6_, ≥99.0%), potassium ferrocyanide trihydrate (K_4_FeC_6_N_6_·3H_2_O, ≥99.0%), and acetone (≥99.5%) was obtained from Sinopharm Chemical Reagent Co., Ltd. (Beijing, China). DIF, bromadiolone (BRD), coumafuryl (CF), and brodifacoum (BRF) were purchased from J and K Scientific (Beijing, China). Phosphate buffered saline (PBS, 0.01 M, pH 7.4) was obtained from Transgen Biotech (Beijing, China). Tetramethylenedisulfotetramine (TETS) and human serum samples were supplied by the Beijing Center for Disease Prevention and Control (Beijing, China) and stored at −20 °C [15]. Nb AR-H2-1 was produced by our lab and will be described elsewhere. Deionized water applied for preparing all the solutions was obtained by Milli-Q^®^ Integral from Millipore (Billerica, MA, USA). In this work, the electrochemical measurements were conducted using CHI760E electrochemical workstations that was purchased by CH Instruments, Inc. (Shanghai, China).

### 2.2. Fabrication and Preparation of the Bioimpedance Sensor

#### 2.2.1. Micro-Nanofabrication of Interdigitated Microelectrode (IDME)

Briefly, silicon dioxide (300 nm) was grown as the insulating layer on the silicon wafer using the thermal oxidation process. After that, Cr/Au (10 nm/90 nm) were deposited with electron beam evaporation that functioned as the seed layer for electroplating. Furthermore, a 1.5-μm-thick photoresist coating (NR1500, Shipley, USA) was spin-coated on the seed layer and then was exposed using an ultra-violet aligner (MA6, Karl Suss) to form IDME. Finally, during the lift-off process, the photoresist was dissolved in acetone, and then the electrode structure was formed. The working area of the fabricated interdigital electrode was 2700 μm by 2000 μm, with the gap space 5 μm. In addition, the amount of the Nbs modification on the polycrystalline gold IDME was compared with the same-sized single-crystal gold IDME. The preparation of the single-crystal gold IDME can be found in previous research [21]. Prior to functionalization, the IDME was pre-cleaned with acetone and distilled water several times and dried in a stream of N_2_.

#### 2.2.2. Preparation of the Bioimpedance Sensor

The polycrystalline gold IDME prepared in this study and the single-crystal gold IDME prepared in the previous study were used to compare the amount of the Nbs attached to the surface of the electrode. Atomic force microscopy (AFM) was employed to characterize the topography modification of the polycrystalline and single-crystal gold IDME after Nb modification. Next, the optimal polycrystalline gold IDME was chosen to establish the bioimpedance sensor. The process of the fabrication of the bioimpedance sensor was conducted as follows. Initially, a 10-μL aliquot of the AR-H2-1 (3.0 mg/mL) was evenly dropped onto the working area of the electrode surface and incubated at room temperature for 2.0 h. In addition, the remaining area of the electrode was covered with a sealing film to prevent contact with the excess Nbs. After successful immobilization of Nbs, the electrode was washed with 1.0 mL deionized water three times to remove the unconjugated Nbs and dried under N_2_ stream. Afterward, the working area of the electrode was incubated in the presence of 10% (*v*/*v* in PBS) calf serum for 1.5 h at room temperature to block probable remaining active sites and prevent non-specific adsorption. The bioimpedance sensor was then obtained after gently washing with distilled water on the electrode surface to remove the redundant calf serum and consequent drying under a gentle stream of N_2_. Finally, the prepared bioimpedance sensor was stored at 4 °C for future use. The surface characterization of the modified gold electrodes was performed using an energy-dispersive spectrometer (EDS) and atomic force microscopy (AFM).

### 2.3. Measurements of the Electrochemical Impedance Spectroscopy (EIS)

The interfacial properties of the gold electrode surface were analyzed using EIS. The electrochemical impedance measurements were conducted on a CHI760E electrochemical workstation. During the process, the circuit impedance variation was analyzed when the amplitude was 5.0 mV, and the applied direct current (DC) bias was set to zero. In addition, the working frequency of 100 Hz to 100 kHz was applied to the IDME. The equivalent circuit was used for fitting the EIS data, and the results are presented in the form of Nyquist plots.

The DIF samples of different concentrations were added to the surface of the bioimpedance sensor, and the sensors’ working area was completely covered by the solution followed by incubation of 10 min at room temperature. After the DIF solution was completely reacted with the Nbs, the bioimpedance sensor was washed with deionized water and blown dry using N_2_. Then, all the impedance experiments were conducted in an electrolyte solution as a redox probe of 10 μL [Fe(CN)_6_]^3−/4−^ (2.0 mM) solution. The impedance measurements of the samples were ordered from low to high concentrations, and the experiments were performed in triplicate.

### 2.4. Analysis of DIF (3-(3-Biphenyl-4-Yl-1,2,3,4-Tetrahydro-L-Naphthyl)-4-Hydroxycoumarin) in the Human Serum Samples

To validate the bioimpedance sensor for real clinical applications, spiked human serum was tested. The human serum was filtered through a 0.22 μm filter membrane prior to use. Then, 10-μL of the DIF standard solution (1000 ng/mL) was spiked into 1.0 mL of a blank human serum sample to prepare the stock solution (10 ng/mL). The stock solution was diluted up to 10-fold with serum to obtain samples with a concentration range of 0.1 pg/mL to 1000 pg/mL. The human serum could be used without particular pretreatment.

To investigate the specificity of the developed Nb-based electrochemical bioimpedance sensor, a series of anticoagulant rodenticides (BRD, BRF, CF) and a type of hetero-adamantane rodenticide (TETS) were chosen to detect the cross-reaction (CR) under the optimized conditions. For this, the bioimpedance sensor was incubated with the rodenticides (100 pg/mL) for 10 min to make sure that the immunoreaction equilibrium was reached. The ΔR_et_ was calculated according to the following equation:

ΔR_et_ = R_et_ (rodenticides reacted with Nb) − R_et_ (control) (1)

where R_et_ (rodenticides reacted with Nb) is the charge-transfer resistivity after immunoreaction with rodenticides; and R_et_ (control) denotes the impedance of the bioimpedance sensor before incubation with the DIF solution. The specificity evaluation was recorded as the CR, and parameters were obtained based on the following equation:

CR = (ΔR_et_ of DIF)/(ΔR_et_ of tested compound) × 100% (2)

## 3. Results and Discussion

### 3.1. Principle and Construction of the Sensor

**Figure 1:**
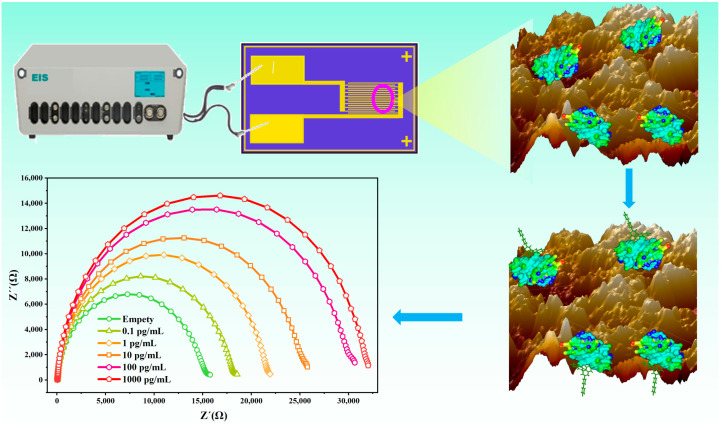
Schematic diagram of ultrasensitive detection of DIF (3-(3-biphenyl-4-yl-1,2,3,4-tetrahydro-l-naphthyl)-4-hydroxycoumarin). The Nb AR-H2-1 was applied to modified on the surface of the electrode to constructed a bioimpedance sensor. Then, DIF solutions were added to the working area of the electrode. After DIF completely reacted with Nbs, [Fe(CN)6]^3−/4−^ (2.0 mM) was added, and the electrochemical impedance were measured on the electrochemical workstation.

The principal scheme of the DIF detection is shown in Figure 1. The Nbs were modified on the surface of the polycrystalline golden electrode and used as the ligand to capture the DIF. It is well known that the number of bio-recognition elements is a significant parameter that influences the property of biosensors. Therefore, to improve the performance of the electrochemical bioimpedance sensor, the polycrystalline gold IDME was fabricated. Then, a novel sensitive Nb-based electrochemical bioimpedance sensor that used a polycrystalline golden electrode for the rapid detection of DIF was established.

### 3.2. Controlled Orientation-Growth of the Gold Nanofilms Electrode

**Figure 2:**
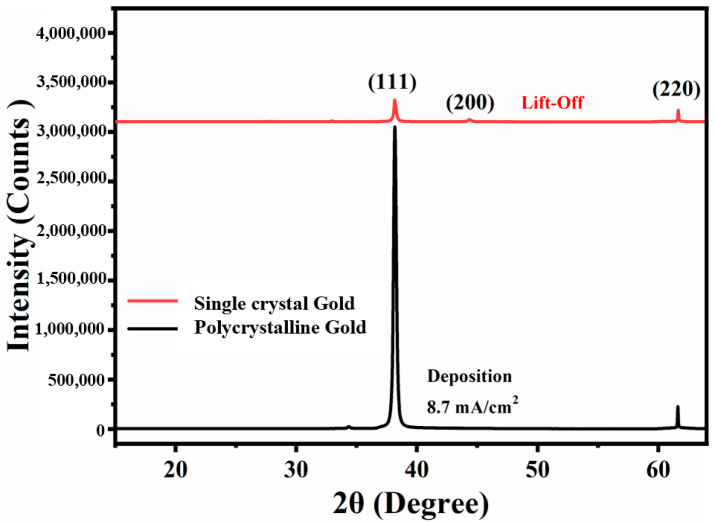
The X-ray diffraction (XRD) spectrum of gold film for lift-off process and deposition processes.

Based on our previous research [21,29], by changing the pulse current density during the electrochemical deposition process, the nucleus growth rate and nucleus density of the gold film on the cathode can be artificially changed to obtain a single-crystal orientation <111> gold electrode. Hence, in this work, the X-ray diffraction analysis (XRD) spectrum of the polycrystalline gold electrode and single-crystal gold electrode were observed. It is clear from Figure 2 that in the polycrystalline gold electrodes prepared using the lift-off process, the crystal orientation of the gold presents the characteristics of the polycrystalline (<111>, <200>, <220>).

**Figure 3:**
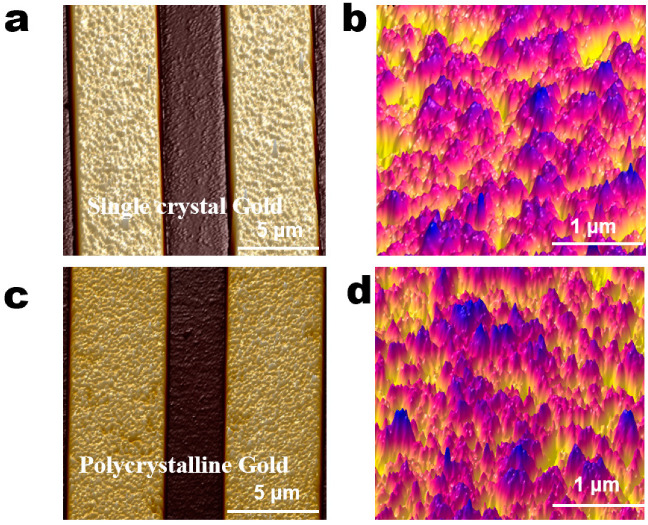
The atomic force microscopy (AFM) images of the single-crystal gold electrode and polycrystalline gold electrode. (**a**) The single-crystal gold film surface (20 μm × 20 μm) was characterized for morphology using AFM; (**b**) surface morphology of single-crystal gold film (3 μm × 3 μm); (**c**) polycrystalline gold film surface (20 μm × 20 μm) was characterized for morphology using AFM; (**d**) surface morphology of polycrystalline gold film (3 μm × 3 μm).

The AFM images can more intuitively represent the micromorphological differences in the gold electrodes with different crystalline grains. The images were analyzed using the Nanoscope analysis 1.9 software (Bruker, Milan, Italy). The bare IDME prepared by an electrochemical deposition process (single-crystal gold) and lift-off (polycrystalline gold) process were characterized. As shown in Figure 3, owing to a change in the manufacture of the IDME, the nucleation and growth mode of the crystal changed such that the morphology of the gold film also showed a marked variation. On electrodes with the same gap size, the surface crystalline grains of the polycrystalline gold film (Figure 3c) were significantly larger than those of the single-crystal gold film (Figure 3a). Additionally, the roughness parameters were obtained by a comparison of the two kinds of IDME. Known from the spectrum of the AFM, the average surface roughness (RMS) of the surface of the manufactured single-crystal gold film was 1.7 nm, and the minimum surface roughness value (Ra) was 1.1 nm (Figure 3b). The RMS of the obtained polycrystalline gold film was elevated significantly up to 7.1 nm, and the Ra was 5.5 nm (Figure 3d). The above results indicated that the surface roughness of the polycrystalline gold electrodes was significantly higher than that of single-crystal gold electrode.

### 3.3. Characteristic and Optimization of the Bioimpedance Sensor

**Figure 4:**
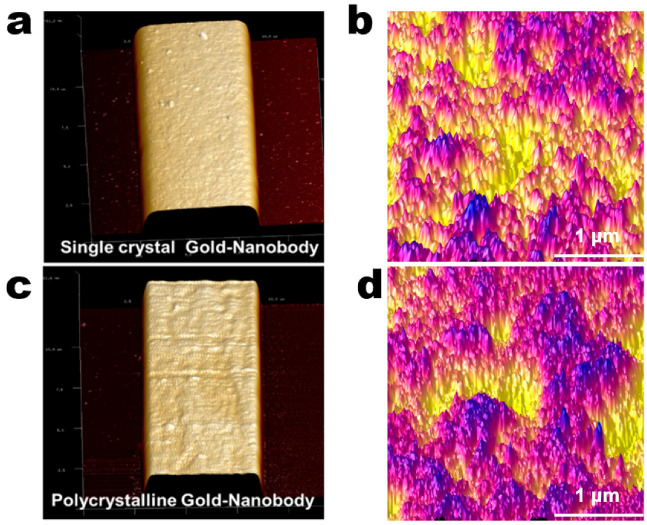
The AFM images of the single-crystal and polycrystalline gold-electrode-modified Nbs. (**a**) Single-crystal gold film (20 μm × 20 μm) was characterized for morphology using AFM; (**b**) surface morphology of single-crystal gold film modified Nbs (3 μm × 3 μm); (**c**) polycrystalline gold film (20 μm × 20 μm) was characterized for morphology using AFM; (**d**) surface morphology of polycrystalline gold film modified Nbs (3 μm × 3 μm).

The purified AR-H2-1 (3.0 mg/mL) was dissolved in the NaAc buffer (0.01 M, pH = 6.0) and immobilized on the surface of the gold electrodes using the Au–S interaction to establish the bioimpedance sensor. To reach the optimal amount of Nbs on the gold electrode surface, the modification capacities of Nbs on the polycrystalline gold electrode and single-crystal gold electrode were compared. The same concentration and volume of Nb were modified on the electrode surface, and then the sensor fabrication was characterized by AFM. Figure 4 shows the AFM micromorphology of the Nbs modified by gold electrodes with different crystal forms. The surface of the single-crystal gold electrode modified Nbs (Figure 4a) was significantly smoother than that of the polycrystalline gold-electrode modified Nbs (Figure 4c). The high-resolution sensing AFM spectrum showed the average roughness of the polycrystalline gold electrode modified Nbs. The RMS of the gold film was 4.6 nm and the Ra was 3.6 nm (Figure 4d), which was higher than that of the single-crystal gold electrode modified Nbs, RMS = 3.7 nm, Ra = 2.9 nm. (Figure 4b). By comparing the blank and Nb-modified single-crystal gold electrodes, the increases in the values of RMS and Rs suggested that the successful immobilization of Nbs will increase the roughness. However, from the roughness factor results of the polycrystalline gold electrode, the RMS and Rs values decreased after the Nbs-modified on the surface of the electrode, indicating the alteration of roughness in the surface morphology. Moreover, this phenomenon can be explained because the nanoscale Nbs can be perfectly modified on the edges and corners of the surface created by the microcrystal clusters of the polycrystalline gold-electrode. Therefore, it was suspected that the polycrystalline gold films provided an increased surface area and would be particularly useful to increase the amount of the Nbs.

**Figure 5:**
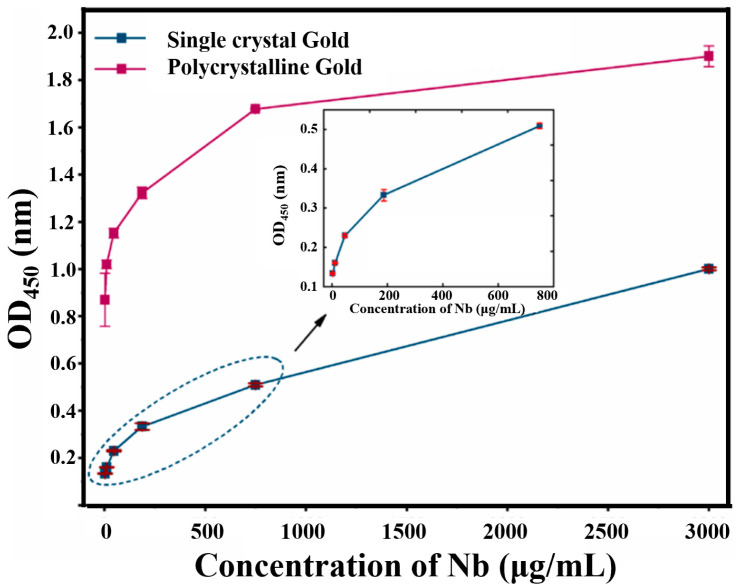
Comparison of the amount of Nbs modification of the polycrystalline and single-crystal electrode. Six different concentrations of AR-H2-1 were immobilized with the two types of electrode. The red line represents the polycrystalline electrode; the blue line represents the single-crystal electrode. The optical density (OD_450_) values of the polycrystalline electrode were higher than those of the single-crystal.

Nevertheless, further study is required to characterize the amount of the modified Nbs on the two electrodes. To prove the coupling effect of polycrystalline and single-crystal gold film, a colorimetric method was performed to compare the Nbs modification amount. The colorimetric results of the two crystal types of the gold electrode are shown in Figure 5. The two types of gold electrode were incubated with different concentrations of AR-H2-1 (four-fold serial dilution, 3000 μg/mL to 0.73 μg/mL). After blocking using 10% calf serum, the anti-HA tag secondary antibody conjugated with horseradish peroxidase (HRP) was used to recognize the Nbs on the electrode. Hence, the amount of Nbs was determined by the HRP activity employing the corresponding substrate 3,3′,5,5′-tetramethylbenzidinefollowby color development for 20 min. In conclusion, the relative optical density (OD) values at 450 nm (OD_450_) of the polycrystalline gold electrode were much higher than that of the single-crystal gold electrode. This result indicated that the polycrystalline gold electrode can provide higher Nbs loading and excellent high-biocompatibility. Therefore, the polycrystalline gold electrode with the maximum binding amount of Nb was selected as a preferable tool to develop a bioimpedance sensor for DIF detection in human serum. In conclusion, it is efficient to attempt such a type of polycrystalline gold IDME for sensitive bioimpedance sensor fabrication.

**Figure 6:**
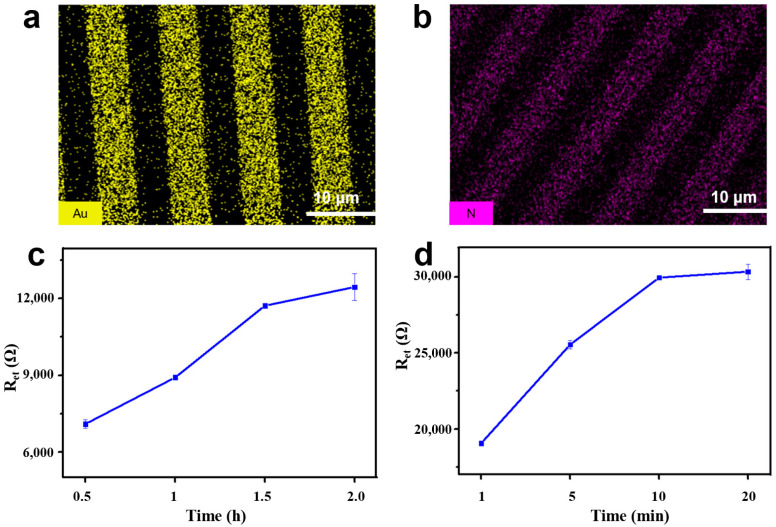
(**a**) The energy-dispersive spectrometry (EDS) spectrum characterization of the bare gold electrode. (**b**) Elemental mapping images of the Nbs modified gold electrode. (**c**) The optimized of immobilization time of the Nb. (**d**) The optimized of the immunoreaction time.

In this study, the electrochemical bioimpedance sensor was proposed by applying a high-affinity Nb (AR-H2-1) to achieve simplicity and the efficient detection of DIF utilizing the combination of Nbs with the classic IDME. Initially, the AR-H2-1 as an alternative recognition element was bonded on the surface of the IDME for the electrochemical detection of the DIF. For this, the polycrystalline gold film surface modified by the immobilized Nbs was characterized. Scanning electron microscopy (SEM) tandem EDS can validate the elements distribution in the gold electrodes. It can be seen that the gold electrodes surface primarily contained Au elements before the Nbs modification (Figure 6a). The existence of the N element was observed in the elemental mapping image (Figure 6b), which confirmed the Nbs modified on the gold electrodes successfully.

For the purpose of achieving the most effective detection performance of the fabricated bioimpedance sensor for DIF, a series of experimental conditions were optimized. For a start, four different immobilization times of the Nb on the surface of the gold electrode were optimized. Figure 6c shows the R_et_ values obtained from each assay. It can be seen that the resistance value increased significantly with an increase in the immobilization time, which reached equilibrium at 2.0 h, demonstrating that the gold electrode was saturated with Nbs. As a result, 2.0 h was chosen as the optimal time for the Nb immobilization time.

Additionally, the effect of the AR-H2-1 immunoreaction time of the DIF exposed to Nb on the surface of the gold IDME was investigated. As shown in Figure 6d, the increase of the incubation time will make for an increase of the captured DIF, which demonstrates an increase in the impedance. It was observed that expanding of the incubation time of the DIF resulted in a maximum response at 10 min. Hence, the immunoreaction time of 10 min was selected for further experiments.

### 3.4. Development of the Bioimpedance Sensor for DIF

**Figure 7:**
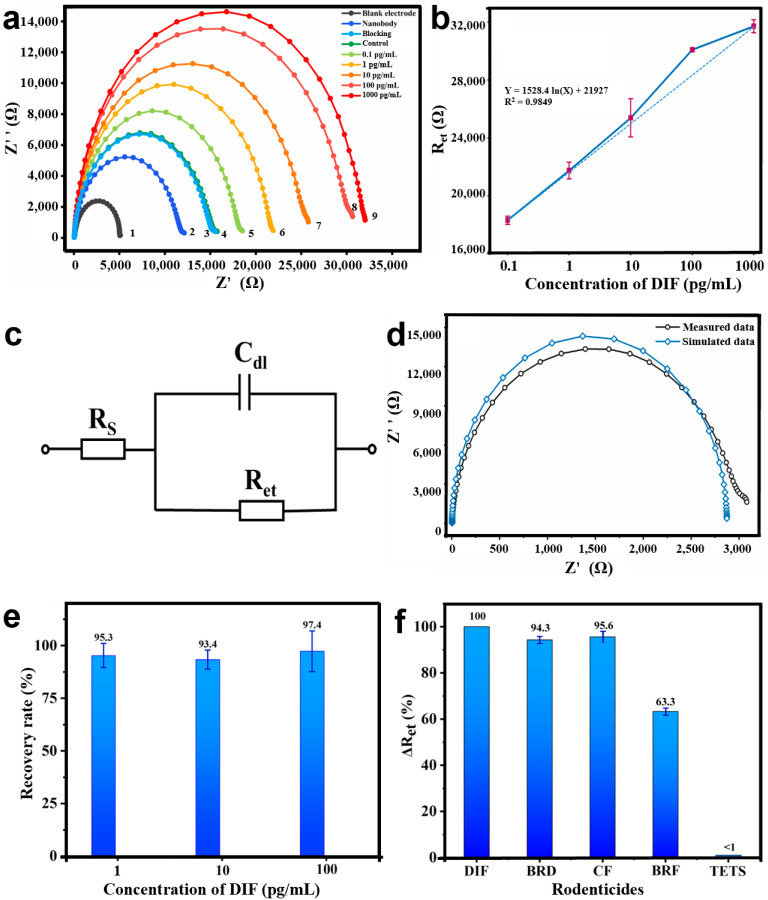
(**a**) The bioimpedance sensor fabrication process and Nyquist diagram for determining of DIF with different concentrations (*N* = 3). Assembling and detection steps of the bioimpedance sensor: Electrochemical impedance spectroscopy (EIS) in 2.0 mM [Fe(CN)6]^3−/4−^ for (1) bare gold electrode, (2) AR-H2-1, (3) calf serum blocking, (4) control. As the DIF concentration increased, more of the DIF was captured by the AR-H2-1 on the surface of the polycrystalline gold film, where the diameter of the Nyquist circle added (5)–(9). (**b**) Linear relationship curve between different DIF concentrations and R_et_. (**c**) The equivalent circuit model used to simulate the working situation of the novel bioimpedance sensor. (**d**) Nyquist plot was demonstrated both in software simulation data (blue line) and actual measurement data (black line) impedance spectra. (**e**) The recoveries of DIF were from 93.4% to 97.4% in serum samples when 1, 10, 100 pg/mL of DIF were added. (**f**) Cross reaction was gained utilizing a series of rodenticides (DIF, BRD, CF, BFR, TETS) at a concentration of 100 pg/mL.

The research shows that antibodies and antigens have poor electrical conductivity, which could prevent the external redox mediator ([Fe(CN)_6_]^3−/4−^) electrons’ transfer toward the electrode surface [20]. The EIS has been demonstrated to analyze the conductivity of the modified electrode. Figure 7 more intuitively displays the scenario when the bioimpedance sensor was used for the detection of DIF. The Nyquist plots show the analysis on behalf of the growth of the charge Ret. The first Nyquist plot semicircle according with the bare IDME revealed a quite low resistance value (Figure 7a, curve 1). After the IDME was modified using Nbs and calf serum, the diameters of the Nyquist plot semi-circles were much larger (Figure 7a, curves 2 and 3). Specifically, the result suggested that the AR-H2-1 and calf serum bonded to the gold electrode’s surface, which hindered the electron transfer processes of the redox couple. The EIS results confirmed the successful assembly of the bioimpedance sensor.

Reducing the serum matrix was necessary to ensure the accuracy and precision of detection before analysis. After the bioimpedance sensor assembly, EIS was employed to display the DIF recognition. It can be seen from Figure 7a, that curves 3 (blocking) and 4 (control) nearly coincide, which revealed there was either no or an insignificant non-specific response detected by the blank human serum. After the Nbs were immobilized on the gold electrodes, AR-H2-1 was used as a potential recognition element for developing a sensitive biosensor to detect the DIF. Moreover, based on the Nbs specific reaction with the DIF, the sum of the non-conducting antigen–antibody complexes increased with the gradual increase in the DIF concentration. In consequence, after incubation of the bioimpedance sensor in different concentrations of DIF for 10 min, the relative impedance of the system further strengthened, further generating the larger semicircles in the Nyquist plots (R_et_). Therefore, from Figure 7a, the diameters of the Nyquist curve increased (curve 5 to 9). The change in the R_et_ values could be used to quantify the concentration of the DIF accurately. These results demonstrated the effective assembly of the bioimpedance sensor and its ability to recognize the analyte.

The DIF concentration (0.1–1000 pg/mL) was used as the abscissa and the impedance values as the ordinate. A linear regression analysis was performed, and a linear equation was developed. The tests were repeated in triplicate to guarantee the duplicability and dependability of the data. As shown in Figure 7b, the Ret responses showed a good linear correlation with the concentration of DIF in the range of 0.1–1000 pg/mL. The linear regression equation could be described as follows: Y = 1528.4 ln(X) + 21927, with a R^2^ value of 0.9849. The LOD (defined as S/N = 3) of the DIF was calculated to be 0.1 pg/mL. Furthermore, the polycrystalline gold electrode with a large surface area provided as many binding sites as possible for the Nbs. Therefore, the EIS characterizations showed that the bioimpedance sensor could be effectively applied to detect the DIF.

In this study, the novel developed bioimpedance sensors were equivalent to the Randle circuit model to simulate the intricate impedance plots with the following elements, as shown in Figure 7c. Typically, R_s_ is the solution resistance, C_dl_ is the constant phase element related to double-layer capacitance, and R_et_ represents the electron transfer resistance (the diameter of the semicircle). Among them, the R_et_ values were susceptible to changes by the electrode surface-modified Nbs, as well as the concentration of the analyte. Therefore, R_et_ was used as the most sensitive element for the DIF analyses.

The Nyquist diagram comparison between the actual DIF measurement data and the software simulation data is shown in Figure 7d. The test curve and the simulation curve nearly coincided, indicating that the equivalent circuit simulation applied to the test work of the bioimpedance sensor.

### 3.5. Application of the Bioimpedance Sensor in Human Serum

To future estimate the potential practical application of the fabricated bioimpedance sensor, detection for DIF was performed by spiking with different concentrations of DIF (1, 10, 100 ng/mL) in the human serum samples. The system recovery efficiency was calculated as a percentage of the detected DIF for the actual added DIF. In this work, the recoveries of DIF were from 93.4% to 97.4% in human serum samples, as shown in Figure 7e, suggesting the bioimpedance sensor was reliable and accurate.

Three anticoagulant rodenticides (BRD, BRF, CF) and a hetero-adamantane rodenticide (TETS) were used to test the specificity of the bioimpedance sensor. The cross-reactivity values were obtained from the average values of three independent experiments. The results are summarized in Figure 7f. The data showed that BRD, CF, and BRF were recognized, with CRs of 94.3%, 95.6%, and 63.3%. In addition, no obvious responses were observed when the electrochemical bioimpedance sensor was in reaction with TETS (<1%). In conclusion, the sensor recognized the most commonly used anticoagulant rodenticides (DIF, BRD, BRF, and CF) which can be treated by vitamin K. Therefore, the bioimpedance sensor can be potentially used to simultaneous screening of a class of anticoagulant rodenticides. In particular, to distinguish between those rodenticides and non-anticoagulant rodenticides.

The bioimpedance sensor stability was studied using EIS in PBS and stored at 4 °C for a week. The EIS measurements were tested by incubating with 10 ng/mL DIF for 10 min at room temperature. The data displayed that the bioimpedance sensor maintained over 85% of activity, representing that the detection system was stable for one week. The results demonstrated the dependability and precision of the bioimpedance sensor and its applicability for the analysis of DIF in human serum.

In addition, the superior features of the current study were highlighted by comparing them with the other published DIF detections in the last five years. The performances (LOD or limits of quantification (LOQ)) are summarized in Table 1. Significantly, among these methods, there was only one study of immunoassays for DIF detection (No. 8). Additionally, the immunoassays detection (No. 8) was achieved by quantum-dot based LFIA with an LOD of 30.6–45.9 ng/mL (ng/g) in serum, urine, and wheat [15]. Nos. 1–7 are all instrument-based detection methods. For instance, as the most sensitive methods proposed by Yan et al., a LOD of ultra-performance liquid chromatography–tandem mass spectrometry (UPLC–MS/MS) for DIF was 0.01 ng/mL [30]. The results confirmed the advantage of the bioimpedance sensor developed in this study with a LOD of 0.1 pg/mL and a detection range of 0.1–1000 pg/mL. It can be declared that as an immunoassay, the sensitivity of the proposed bioimpedance sensor was the highest, which is beneficial for the rapid and sensitive detection of DIF in human serum samples.

## 4. Conclusions

In summary, a Nbs-based bioimpedance sensor for ultra-sensitive trace detection of DIF in human serum was developed. In this study, the polycrystalline gold electrode provide a larger surface area, outstanding biocompatibility, and a high Nbs coupling rate. Subsequently, the developed bioimpedance sensor constructed in this study provided a high sensitivity LOD of 0.1 pg/mL and wide linear range for DIF quantification, which is a better performance than that of other methods. Additionally, the recovery and stability demonstrated the accuracy, precision, and stability of the bioimpedance sensor. Moreover, the developed bioimpedance sensor have a broad specificity which has the potential applications of diagnosis of extensively used anticoagulant rodenticides. In conclusion, the proposed detection platform was sufficiently sensitive to meet the requirements for the early diagnosis and monitoring of DIF in human serum samples and possesses promising applications for the determination of other analyses.

## Figures and Tables

**Table 1:** Comparison of the detection results of DIF with other published research in the last five years.

No.	Method of Detection	Detection Results	Sample	Ref
1	UPLC–MS/MS ^a^	LOD: 0.01 ng/mL, linear range: 0.1–100 ng/mL	Urine	[30]
2	LC–MS/MS ^b^	LOD ^c^: 0.2 ng/mL, linear range: 0.5–50 ng/mL	Blood	[31]
3	UHPLC–MS/MS ^d^	LOQ ^e^: 1.5–2.7 ng/g, linear range: 2.2–1111 ng/mL	Faeces	[32]
4	UPLC–QTrap-MS/MS ^f^	LOD: 0.02 ng/mL, linear range: 0.1–100 ng/mL	Food	[33]
5	UPLC–MS/MS	LOD: 0.75–25 ng/g, linear range: 50−2500 ng/g	Liver	[34]
6	UPLC–MS/MS	LOD: 0.25 ng/mL, linear range: 1–2000 ng/mL	Blood	[35]
7	UHPLC ^g^	LOQ: 0.2 ng/g, linear range: 0.2–150 ng/g	Liver	[36]
8	QNs–based LFIA ^h^	LOD: 30.6–45.9 (ng/mL, ng/g), linear range: -	Serum, Urine, Wheat	[15]
9	EIS	LOD: 0.1 pg/mL, linear range: 0.1–1000 pg/mL	Serum	This study

^a^ ultra-performance liquid chromatography-tandem mass spectrometry, ^b^ liquid chromatography-tandem mass spectrometry, ^c^ limit of detection, ^d^ ultra-high performance liquid chromatography-tandem mass spectrometry, ^e^ limits of quantification, ^f^ ultra-performance liquid chromatography-triple quadrupole/linear ion trap tandem mass spectrometry, ^g^ ultra-performance liquid chromatography, ^h^ Quantum dots based lateral flow immunoassay (LFIA).

## Data Availability

The data presented in this study are available on request from the corresponding author.
